# Exploring the transformative power of AI in art through a circular economy lens. A systematic literature review

**DOI:** 10.1016/j.heliyon.2024.e25388

**Published:** 2024-02-02

**Authors:** Pedro Núñez-Cacho, Georgios Mylonas, Athanasios Kalogeras, Valentín Molina-Moreno

**Affiliations:** a Department of Business Organization. University of Jaen, Jaén, Spain; b Industrial Systems Institute, Athena Research Center, Patras, Greece; c Department of Management, University of Granada, Granada, Spain

**Keywords:** Art, Artificial Intelligence, Circular Economy, Review, Algorithms

## Abstract

Today, technology and sustainability are two strategic axes for the development of any industry. Art is no exception and embodies both principles. Artificial intelligence (AI) is driving the art world forwards with its applications and algorithms. Additionally, the circular economy (CE) is concerned with resources and the environment in this context. The objective of the present work is to provide an overview of the current state of research on the application of AI in the art world and an analysis of how CE principles are being incorporated, considering the interactions between AI and the CE. To this end, a systematic review of the literature is carried out in which 60 articles related to the subject are selected, analysed, and classified, highlighting the lines of research addressed. The assessment of the current state of research on the subject concludes with the four main axes of classification of works. The first line is related to AI generative content in art, addressing issues of content creation, image and painting, video, and theatre. The second line is related to AI applications for art industry production, considering the sustainability of the supply chain. The third line focuses on how the CE is being applied to art, while the fourth line focuses on other relevant aspects analysed, such as training and design. The topic is still incipient, mandating further research to study the full potential of AI and the CE in the world of art.

## Highlights

• The paper provides an overview of the state of research on the application of AI in the Art world and an analysis of how CE principles are being incorporated, considering the interactions between AI and the CE.
• The assessment concludes with the four main axes of classification of works. The first line is related to artistic creation through AI, the second is related to AI applications for Art industry production, the third focuses on how the CE is being applied to Art, while the fourth focuses on other relevant aspects analysed, such as training and design.
• The topic is still incipient, mandating further research to study the full potential of AI and the CE in the world of Art.

## 1 Introduction

Today, we live in a technological society, where AI [1] and other emerging technologies, such as cloud computing, the Internet of Things or IoT [2], digital twins [3,4] and blockchain [5], are being incorporated into daily lives at great speed, making it critical to understand the processes that are carried out [6,7]. AI represents one of the most promising paradigms [8,9], comprising systems that have analysis capabilities capable of emulating human cognition [[10], [11], [12]]. The technology is imposing itself in different sectors of society, business, and life in general, and its application is following exponential development in various topics, including design, transportation systems, nanoscience, biotechnology, education, sustainability, etc. [[13], [14], [15]].

In the context of this work, art is understood as any activity or product carried out with an aesthetic and communicative purpose, through which ideas, emotions and, in general, a vision of the world are expressed through various material and resources, including plastics, language, sound, body and mixed media [16]. Art is not exempt from the use of information technologies in general and AI in particular [13]. For instance, robotics have been used in the artistic creation of paintings [11,12,17], big data and machine learning have been used in product design [18,19], and the Internet of Things [20,21] and blockchain have shown effectiveness in improving the traceability of works of art [22,23]. In this sense, AI can have multiple applications in artistic manifestations [24], yet research works that address this issue (the intersection of AI and art) are very recent, as art has traditionally been associated with human activity and social sciences. Currently, there are no pieces of research focusing on analysing and synthesizing existing scientific contributions in this field.

On the other hand, we are at a time when society has become aware of the problem of environmental sustainability [21, 25] The continuous increase in the world population and the changes in economic and social models with the negative externalities generated have resulted in a situation that requires immediate action and exerts intense pressure on companies to stop generating such externalities [26].

All this acts as a disruptor for companies, which, in their eagerness to survive, seek to reduce their carbon footprint and adopt a more sustainable economic model [23]. In this context, the circular economy (CE) presents a vehicle that could facilitate the achievement of such objectives. The CE is defined as a restorative and regenerative economy by intention and design and marks the end of the linear economy model, which began with the industrial revolution and continues to this day [27]. Sustainability and the CE are currently supported by technological elements that help achieve their planned objectives. Currently, we are at a historical moment in which the new technology models are trying to address the challenges posed by sustainability. At this point of convergence, AI and the CE meet and coexist in today's organizations [10, 28]. The number of research works related to the interactions between information technologies and art is growing. Science, art, and technology have been linked since the 1960s, when scientists, artists, and innovators began to collaborate and use electronic instruments to produce art [24], and electronic media is ushering in a new way of making art [6, 23, 29]. In this context, the objective of the present work is to provide a global vision of the current state of research in the intersection of the fields of AI, art, and the CE.

The methodology followed to achieve this objective is that of a systematic review of the literature (SLR). According to Xiao & Watson [30], to advance knowledge, we need to define its current frontier. This will allow us to explore gaps and develop new theories. The SLR methodology allows the identification, selection, and critical analysis of contributions to the literature on a specific research topic [31,32]. Thanks to this knowledge, researchers can establish the principles that require new theoretical and conceptual models in a study area. An SLR requires three stages: planning, carrying out, and reporting research [31,33].

The research questions of this work are as follows:

**RQ**. How is AI being applied in the world of art, and what is its contribution to the CE?

RQ1 What categories of articles have been published to date?

RQ2 Is it possible to identify a taxonomy of current research on AI, art, and the CE?

RQ3 What gaps exist in the literature on AI, art, and the CE, and what lines of research should be developed in the future?

Regarding the structure of this article, after this introductory section, the rest of this paper is organized as follows: Section 2 reports the proposed SLR based on co-occurrence and content analysis. The results and discussions are presented in Section 3. A full analysis of the articles reviewed highlighting the relationships between the risk measures, decisions, the manufacturer's risk attitude, and the modelling techniques used are also reported in Section 3. Finally, research gaps and future research directions are highlighted in Section 4.

## 2 Conceptualisation: Artificial intelligence, art, and the circular economy

### 2.1 Art and the CE

Art is a diverse range of human activity generating a result that involves creative or imaginative talent and that displays technical skill, beauty, emotional power, or conceptual ideas. There is no generally accepted definition of what constitutes art [34,35], and its interpretation has varied throughout history and across cultures. The three classic branches of visual arts are painting [7,36], sculpture, and architecture [37]. Theatre, dance, and other performing arts, as well as literature, music, film, and other media, including interactive media, constitute a broader definition of the arts.

The CE and sustainability are fundamental concepts from a scientific perspective and, in the context of the art world, have several significant implications. The production and exhibition of works of art often involve the use of natural materials and resources; this is known as ecological art, where the art promotes environmental protection [38]. Thus, it is necessary to integrate the CE into artistic and craft creation [39], because it can considerably reduce the environmental impact of the emission of greenhouse gases during artistic creation processes, energy consumption, and other negative environmental externalities. To achieve this, the CE seeks artistic production with new designs [40], based on the use of renewable energy, efficient technologies, and cleaner production processes that reduce the use of chemicals. In this way, resource extraction is reduced, and waste generation is minimized [41].

Furthermore, throughout the life cycle of an artistic work, its maintenance and preservation are also subject to sustainability criteria. The use of sustainable materials and techniques in the restoration and conservation of works of art contributes to their longevity and prevents irreversible damage. It is important to highlight the role of art in communicating messages and raising social awareness of fundamental issues related to the CE. When artists adopt CE practices, they convey a positive message about the importance of caring for the environment and living more sustainably [39].

Another notable aspect is that the CE can drive innovation and creativity in the art world, challenging artists and creators to find new and creative ways to use materials, reduce and recycle, and address environmental issues in their work. This can lead to the creation of unique and meaningful works of art, generating impactful pieces that use circular and regenerative materials that can be reintroduced into the system [42], or creatively reused, which in turn can inspire others to follow that example. It can also give rise to new business models for artists, generating a sustainable entrepreneurship model [43].

### 2.2 Art and IA

The relationship between art and technology has been the subject of study and analysis in fields such as art history, aesthetics, media theory, the history of technology and the history of science. In the Industrial Revolution of the 19th century, technology began to have a significant impact on artistic production with the appearance of photography, which allowed a precise and objective representation of reality, influencing painting and sculpture. At that time, new materials and techniques supported by the Industrial Revolution were developed, such as printing and metallurgy, with effects on the visual and decorative arts.

In the 20th century, technological advances such as radio, television, and film created new forms of art and entertainment. This sparked the interest of contemporary artists in the possibilities of using media and technology to express ideas and emotions in innovative ways. In 1919, the Bauhaus project, which promoted the development of new avant-garde artistic models, was created. In the last decades of the 20th century, the Institute of Contemporary Art in London promoted the first exhibition of art and technology, promoting collaboration between engineers and artists.

With the development of computing, the Internet and digital technology, new forms of art have emerged. Artists have begun to use software, hardware and networks to create works of art that explore the relationship between humans and machines, with purposes ranging from assisting in the design process to analysing material pigments in restoration, conservation, and interpretation [44]. The appearance of the Internet has also driven changes in artistic marketing and exhibition models, resulting in a wide field of tools at the service of artists. We can group the results of this development into two categories: technological tools and AI generative content (AIGC) technologies.

Regarding the tools, we find virtual reality and augmented reality useful for presenting interactive works of art and unique experiences for the viewer, and they are also linked to the metaverse, such as the 3D art gallery [45]. 3D printing is a versatile tool for artists, allowing them to create three-dimensional sculptures and custom works of art from a wide range of materials. Graphic design programs and image editing software allow artists to create digital works, including illustrations, digital paintings, and pixel art. New digital photography programs allow artists to crop and manipulate images or to regenerate existing ones. Additionally, the presentation of art online and the use of social networks to exhibit and promote works of art facilitate the dissemination of works to a global audience. Also of great interest are digital techniques applied to the conservation of works of art [46] or to the analysis and interpretation of specific aspects of the works [7].

AIGC is undoubtedly the most disruptive approach and represents the greatest challenge for the art world. AI is used to autonomously create art or help artists create works, such as generating music, writing poetry, and painting. Generative content in art refers to art created by a system that operates autonomously [46]. AIGC develops algorithms and models capable of generating synthetic data that resemble aspects of the real world [47]. Programming and artificial intelligence can be used to create generative art based on these algorithms and automatic processes that produce works of art in constant change and evolution [48]. They also pose a new challenge for artists regarding the authorship of their own works, a debate that has arisen in particular since the auction of the AI-generated Edmond de Belamy painting [49].

The nature of art and related concepts, such as creativity and interpretation, are explored in a branch of philosophy known as aesthetics [50]. The resulting artworks are studied in the professional fields of art criticism and art history. In this context, creativity is a characteristic or process that results in the production of some novelty that can be tangible or intangible [51]. Human creativity is exploratory, combinatorial, and transformational in nature [52]. The more transformative creativity is in the process of creation, the greater the surprise and value placed on it [49]. Systems that incorporate AI possess analytical capabilities that emulate human cognition. According to Nilsson [53] AI is an activity dedicated to making intelligent machines, while intelligence represents the quality that allows an entity to function properly and with foresight in its environment.

**Fig. 1:**
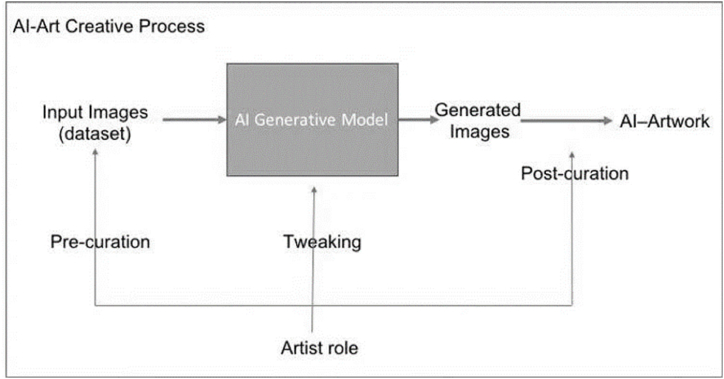
A block diagram showing the artist's role using the AI generative model in making art. Source: Elgammal & Mazzone [55].

One of its differentiating features is machine learning, with three main branches: supervised, unsupervised, and reinforced learning. Supervised learning, which is the least autonomous method, uses expert knowledge to verify hypotheses derived from data analysis [19,54]. Previously, it involved humans, who predefined and labelled the data used. The application of AI to art follows a process such as the one shown in Fig. 1.

### 2.3 Preliminary reviews on the subject

The concept of the CE arises as a set of ideas drawn from different economic and environmental approaches, the main objective of which is to improve the efficiency of resources and strike a balance between the economy, the environment, and society [[56], [57], [58]]. Stahel [59] pointed out the need for a change in thinking from property to use. In recent times, new key ideas have been incorporated, such as the cradle-to-cradle theoretical concept [60], ecology, cycle and yield economics, regenerative design [61], biomimetics [62] and the blue economy [63,64]. Following the principles of CE, redesign is based on the ability of companies to use products and services more intensively and develop awareness among stakeholders, emphasizing the importance of minimizing the use of natural resources [65]. This encourages the reuse of products that still present operational functionalities, favouring the development of repair systems [66]. At the same time, companies consider remanufacturing and repairing, extending the useful lifecycle of products, both for their use and to develop different functionalities [67,68]. At the same time, the most efficient use of recycling, considering circular principles, allows energy and materials to be recovered, either to incorporate them back into the cycle or to favour natural absorption [69].

## 3 Methodology

### 3.1 Introduction

**Fig. 2:**
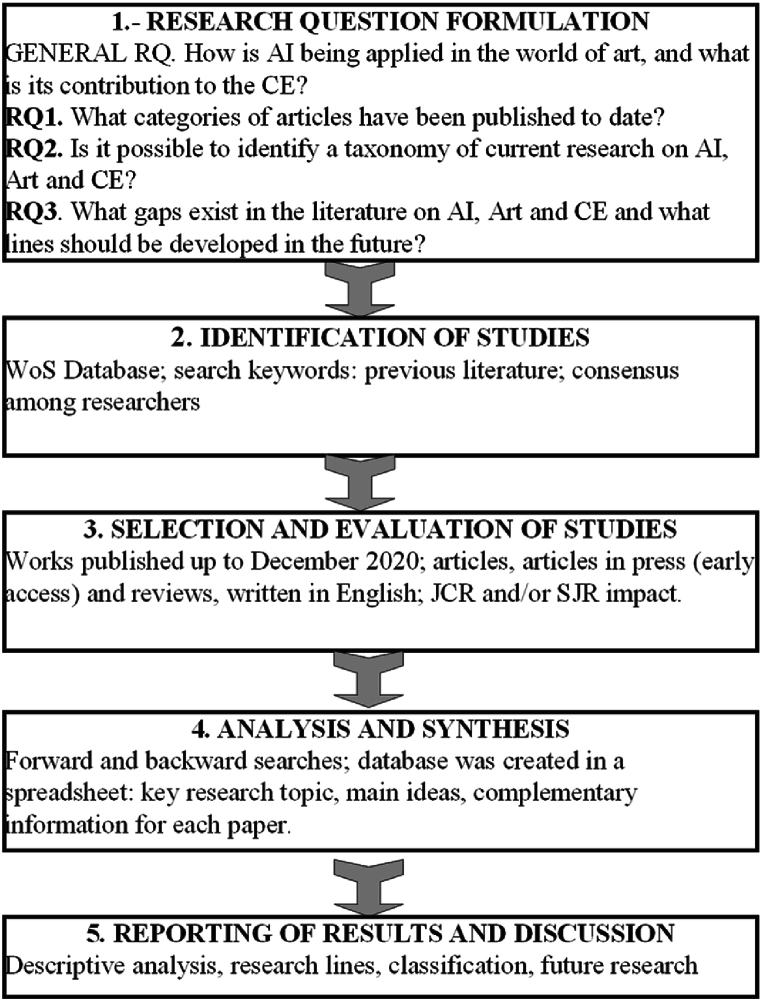
Phases of SLR. Source: Denyer & Tranfield [32].

We present an SLR for the intersection of AI, art, and the CE based on co-occurrence and content analysis, addressing a relevant gap in the literature. The SLR allows us to structure a research topic, determine the state of research of a subject, and map current studies [70]. Its development follows a series of steps according to an established procedure that allows the replication and verification of the results [31,32]. This methodology (SLR) is suitable for the objectives of detecting the use of AI technologies and their relations with art in a CE context. For the development of the technique, we follow the steps proposed by Denyer and Tranfield (see Fig. 2).

### 3.2 Phase 1: research question formulation

During this planning stage, we identified the research needs in the field of the intersection of AI, art, and the CE. Then, there was a more extensive identification of goals and research questions to cover the gaps detected in the relationships between AI, art, and the CE and to establish the theoretical base linking these concepts. We then proposed research questions that would drive the research process [30,33] and developed a review protocol.

### 3.3 Phase 2: study location

**Fig. 3:**
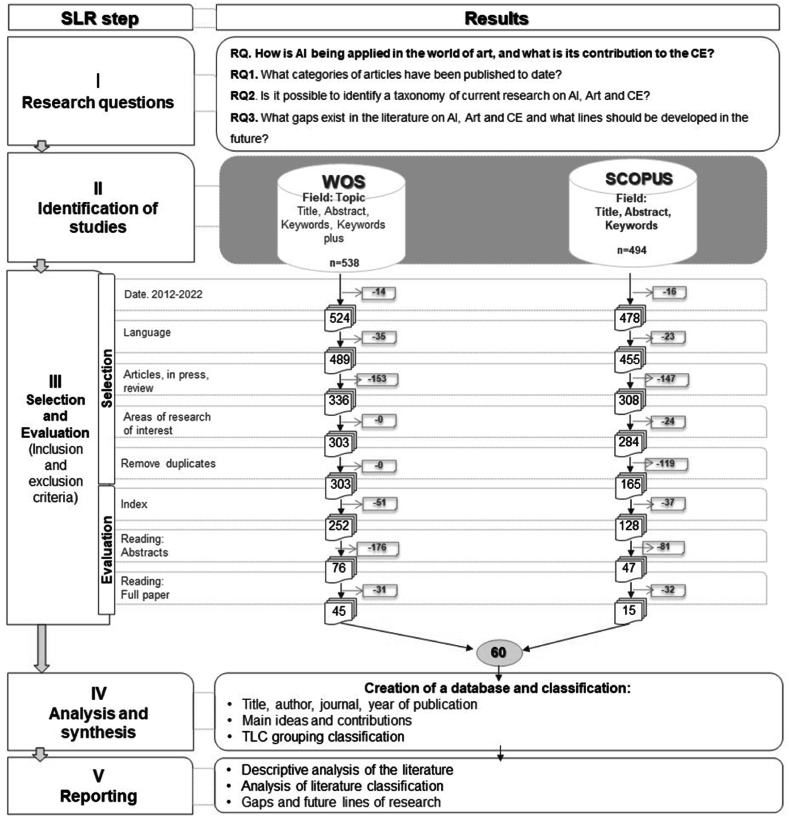
Phases of PRISMA methodology. Source: Adapted from Maqueira-Marín et al. [72].

Once the research questions have been established, the next step, according to the Denyer and Transfield [32] model, is to locate the most significant studies related to RQ. To do this, we addressed two key questions: which search engines and search strings to use. Regarding the first, we chose two databases widely used in the review studies: (i) the Web of Science (WOS woscc.fecyt.es): Web of Science (WoS) is a recognized scientific database and is widely used as a search engine for SLR; (ii) the Scopus database (https://scopus.com) was also used, being recognized in the literature as suitable for this type of work. The objective of the research is to study the intersection between AI, art, and the CE. An analysis of search strings in previous studies e.g., Novais et al. [71]; Maqueira et al. [72] and a brainstorming session among the authors helped identify search keywords related to the research objective. A search string was created using simple operators, including truncated characters (e.g., *, “exact phrase”) and Boolean operators (e.g., AND, OR). The “subject” (TS) search field available in WoS was determined as the tag of searches, which means that the defined sets of keywords must appear in the title, abstract, article keywords, or keywords plus. This process identified 1032 documents (see Fig. 3). The final search strings made up of the previously defined keywords were built:

[(“ART”) OR (“ART MAKERS, CRAFT, ARTWORK, HANDICRAFTSMEN”) AND (“ARTIFICIAL INTELLIGENCE” OR “IA” OR “AI” OR “MACHINE LEARNING”) AND (“CIRCULAR ECONOMY” OR “CIRCULAR” OR “SUSTAINABILITY” OR “ECO")]

### 3.4 Phase 3: study selection and evaluation

Once the search was carried out, a total of 1032 research articles, preprints, reviews, and conferences indexed in WoS and Scopus were found according to the inclusion criteria (selection phase); that is, the article included in its title, keyword or abstract the words established in the search string. The objective pursued in the evaluation phase is to discard those studies that are not relevant to the object of the investigation. To do so, the exclusion criteria were defined [32,72,73]. Fig. 3 shows the papers that were eliminated in each step and those that remained after applying each filter. After these criteria were applied, articles that did not belong to the review dataset were eliminated, and those that could be included were selected. Fig. 3 shows a PRISMA (preferred reporting items for systematic reviews and meta-analyses) flow diagram [74] to illustrate in detail the different phases established for the SLR followed in this study.

### 3.5 Phases 4 and 5: analysis and synthesis phase and report of results and discussion

During this phase, the research team proceeded to the analysis and synthesis of the articles selected in phase 3. With this information, a database was created, and the most relevant information was codified in a structured manner, with the participation of multiple researchers in the process [32,74] to minimize single investigator bias and ensure the reliability and validity of the results.

## 4 Results

### 4.1 Descriptive results

The academic literature that has addressed the study of the intersection of AI, art, and the CE is growing. Relevant research was enhanced during the analysis period. On the other hand, within the typology of selected articles, a predominance of literature reviews is observed, being an indicator of growth stage for the studied disciplines.

**Fig. 4:**
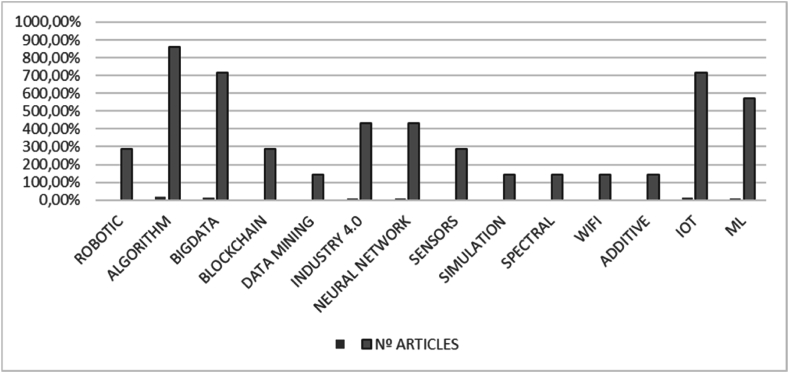
Articles by side technology.

The effort of the researchers to identify the types of algorithms applicable to the phenomenon under analysis can be observed (see Fig. 4). Big Data also occupies a prominent position next to machine learning, highlighting the importance of data management and learning. The contribution of the Internet of Things to art and the CE should also be highlighted, being an object of analysis by a considerable number of authors.

Regarding the journals in which the publications have appeared, there is a great dispersion, with articles from different journals that have published a sparse number of articles focusing on the topics of the review being collected. This could be indicative and a consequence of the multidisciplinary nature of the topics that were analysed in the review: AI, art, and the CE. Some journals stand out in this list: Sustainability, for the CE aspect, with five published articles; Computers & Industrial Engineering, about AI and technology, with four published articles; and Artnodes and The Design Journal within the field of the arts, with two publications each.

### 4.2 Analysis of contents and classifications

**Fig. 5:**
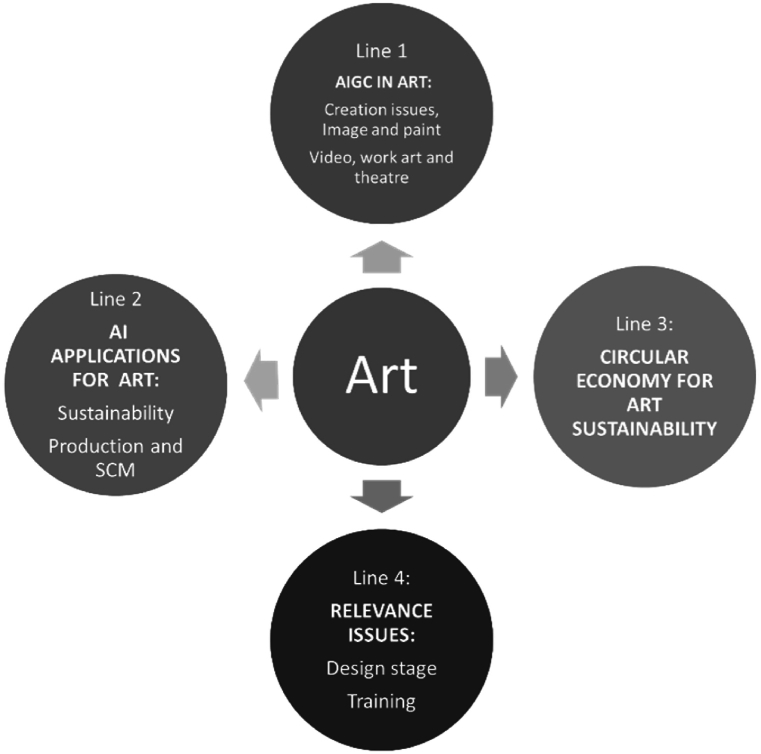
Main research lines identified.

We summarize the main research lines identified in Fig. 5 and Table 1.

**Table 1:** Research lines identified.

RESEARCH LINE	ARTICLES
**LINE 1** **AIGC IN ART** **Creation issues, image, and paint, video, and theatre**	[9,12,13,[17], [18], [55],^36^,^49^,[75], [76], [77],[78], [79], [80], [81], [82], [83]]
**LINE 2** **AI APPLICATIONS FOR ART** **Technologies, sustainability, production, and SCM**	[6,7,10,14,19,21,23,65,67,[84], [85], [86], [87], [88], [89], [90], [91],[92], [93], [94], [95],^96^]
**LINE 3** **CIRCULAR ECONOMY FOR ART SUSTAINABILITY**	[1,2,15,20,39,41,80,97,[98], [99], [100], [101], [102], [103], [104]]
**LINE 4** **RELEVANCE ISSUES** **Training, design stages**	[40,101,[105], [106], [107], [108], [109]]

#### 4.2.1 Line 1: artificial intelligence generative content in art

The application of technology, big data, machine learning and AI in artistic works has intensified in recent times [18] promoting the autonomous process of content generation through AI. The changes introduced by AIGC generating synthetic data that resemble aspects of the real world [47]. AI can be used to create generative art based on these algorithms and automatic processes that produce works of art in constant change and evolution [48]. AI is used to autonomously create art or help artists create works, such as generating music, writing poetry, and painting. Generative content in art refers to art created by a system that operates autonomously [46]. It is true that art, with a key component of human creativity, is one of the fields that is a priori regarded as less conducive to technology. Can a computer create art? This dilemma was posed by Maiocchi [11] more than three decades ago, and there is a need to provide an answer to it by analysing the most relevant approaches in the study of perception and creation of computer art, focusing on results obtained in artistic infographics. Maiocchi pointed out that there was still a long way to go. Recently, the same question has been raised [77] affirming this possibility in the case that one day AI is developed with intelligence and consciousness at the human level. However, making predictions about a true AI is regarded as impossible [110], with the author not believing that AI can reach the social component that art includes. Finally, Stanley [9] addresses the issue of creating art, noting that scientists have spent years looking at nature and thinking about its connection to computing. He notes that feeling is behind all inspiration and that the spark of inspiration can never be ignited from one scientist to another unless by completely objective means. He doubts that this can make sense in fields based on the artificial interpretation of reality. A further interesting contribution is the research of Sun et al. [36], which compares, in a sample of 380 participants, 6 works generated by AI with 6 works by an amateur author, highlighting the limited effectiveness of AI in modifying an existing style in a painting and arguing that the works produced by AI are based on technology, while human works are based on emotions.

Algorithms for image and paint

Generative content in art refers to art created by a system that operates autonomously [46]. The availability of data in different formats (text, images, or sounds), machine learning, and the development of algorithms have opened a new path in the development of the art world [77]. points out that technologies offer new opportunities to artists. Table 2 shows the main algorithms applied to art, highlighted in the review articles. Algorithms in AI are conceived as a tool for the artist, and innovative technologies help keep art alive and do not mean a loss of employment. An example of this utility is the research by Guo et al. [7], who applied visual neural network algorithms to intelligently analyse the emotional expressions that appear in oil paintings, a painting format with very little application of technology. Future AI developments will be future tools for artists. Fortuna & Modliński [49] state that the paintings made by AI are not perceived as the same as those created by humans. However, the experiment [75] that sought to determine if people can differentiate between works created by humans and those created by machines concluded that they could not be differentiated accurately and that, as a rule, people associate machines with more abstract art. Nevertheless, the first portrait created by AI (Edmond Belamy) has already been auctioned at Sotheby's [49]. In this regard, Epstein et al. [17] point out that no AI acts alone, completely divorced from the influence of humans. The authors ask two questions: How do people think credit and responsibility should be assigned to the various actors in art production through AI? How do these intuitions vary according to people's perceptions of the anthropomorphic nature of the AI system? The authors analyse the chain of ownership of the works and in their conclusions point out that credit must be given to the programmers in the creation of art.

**Table 2:** Algorithms used in the articles on RSL.

ALGORITHM	USE	AUTHORS
Neural networks	Spectral training	[78]
ENVI SHW (Spectral hourglass Wizard)	Spectral	
Craig Reynolds' Boids Algorithm	Images	[88]
NEAT (Neuro Evolution of augmenting images)	Images	
GAN (General Adversarial Network)-Like algorithms; X-GAN, Y-GAN, Z-GAN	Artwork, Images	[55,17,75]
Playform algorithms	Images	[55]
Variational autoencoders (VAEs),	Video	[82]
Non-Photorealistic Rendering (NPR) algorithms	Image Creation	[109]
Painterly rendering algorithms	Image Creation	
A* Algorithm	Artwork	[7]
Genetic Algorithms	Artwork/Disassembly	[11]
LS (Link State) system	Generative Design	[76,92,108]
Swarm intelligence (SI) algorithms; Particle swarm optimization	Art creation/Design	
Simulation Localization and Mapping (SLAM)	Art	
Shape grammars, Generative design algorithms	Generative design	
Branch-and-bound algorithms	Disassembly	[14]
K-means clustering algorithms	Classification	
Optimization algorithms	Repair/Reuse	[92]

#### 4.2.2 Line 2: AI applications for art

Undoubtedly, AI-based technologies are useful for sorting waste at plants [80]. Among the advances in AI and deep learning [78], the improvement in image classification systems can allow the identification of waste for automatic recycling, using convolutional neural networks that would provide the process with greater capacity and precision than human interaction. For this purpose, a process based on image capture is developed. Subsequently, using machine learning in the cloud, a model is generated that classifies the object for its next reuse destination with 90 % reliability. We are thus facing a promoter of the CE [85]. It also addresses the analysis of waste with sensors, proposing that an AI system with multiple directed sensors analyses plastic waste and classifies it into different types, assigning it the appropriate process for its treatment [23,84]. It is important to highlight the role that AI can play in this design phase [10, 88] and in the final phase during the disassembly of industrial products [14]. The search for a design optimized for sustainability, therefore, becomes one of the priorities of the research. Among the tools for this purpose, we find pinch analysis, known for its efficiency in reducing the use of resources through thermodynamic concepts [88].

Another outstanding tool is the process graph, developed by Friedler et al. [71], which is used to solve network-type optimization problems and combine algorithms such as combinatorial solution search and branch-and-bound. AI with process analytical networks, computer-aided modelling, and lab-scale studies including 3D printing are the fundamental elements in this new design model [88].

Asif et al. [97] point out that technology plays a determining role in the transition towards the CE, supporting the development of products as a service and sharing information in real time. Waltersmann et al. [19] analyse through an SLR the role of AI in aspects related to sustainability, identifying a clear gap in its application to the efficient use of resources. Among the AI tasks applied to resource management are trend analysis, classification, anomaly detection, image recognition, modulation of language processes, clustering, dimension reduction, and learning tasks. Methods with potential include support vector machines, convolutional neural networks, pattern recognition, recurrent neural networks, and the long short-term memory model. The application of AI shows great potential in the efficient management of energy, emissions, and materials. In addition, the principles of thermodynamics, entropy, masses, heat transfer, etc., need to be considered in the production processes under the CE paradigm. Waltersmann et al. [19] note the following as challenges of digitalization: the definition of technological processes and platforms, the management of thermodynamic data, the transfer of heat and mass in the reactions that occur, and the digitalization of processes and their control [91].

Smart product data handling has high potential for improving product performance, reliability, end-of-life treatment, and energy consumption. In this way, predictive capabilities are generated that allow anticipating the processes and managing to generate less waste, a key concept in sustainability analysis [20]. The information provided by the smart product during its useful life will make it possible to improve product characteristics, favouring its reintroduction into the cycle, which is a goal of the CE [87,90].

Production systems and the SCM of art

The transformation of production systems entails financial decisions and requires a cost analysis of the processes. Chen et al. [86] propose a methodology for this task based on the application of AI, fuzzy knowledge, fuzzy logic, and genetic algorithms. The calculation of environmental costs is obtained employing a supervised learning algorithm such as the decision tree. The target variable/outcome is predicted from a group of predictor variables. Undoubtedly, the issue of costs is vital for decision-making in the transition scenario towards the CE. The RECLAIM project also develops an analysis tool for intelligent decision-making [94] when calculating repair and maintenance costs during the useful life of the machinery. In this context, technologies and innovation are strategic concepts [89]. Aziz et al. [92] point to the current limitations in applying AI to optimize the design so that the generated products are reusable and repairable. These authors also highlight the usefulness of additive manufacturing for these processes. Certainly, technologies such as the IoT show great potential in the CE; an example of this is the application of labels with QR codes and sensors to generate a product passport that allows the circular supply chain, as stated by Gligoric et al. [2]. This would be the essential reverse logistics for the development of the CE [95]. On the other hand, Kerin & Truong [67] highlight labelling products to identify them, follow their traceability, and detect events within the production environment focused on I 4.0. In this way, IoT technologies are being increasingly used in sustainable production environments [21].

The sustainable supply chain supported by AI is another topic that we frequently find in this SLR. Zacharaky et al. [94] formulate a conceptual framework that integrates AI and sustainability and analyses the supply chain from five perspectives: business, technology (Internet of Things, Industry 4.0), sustainability, collaboration, and strategy. In their model, they develop the reverse supply chain as the fundamental axis of sustainability, to which the literature is paying more attention every day [21, 25, 67, 95, 96].

Within the concern of innovating in the production system, data mining presents its potential for the application of disassembly techniques to measure process times. Moreover, the work addresses the role of AI driven by data analytics and big data to improve the supply chain [14,93].

Jabbour et al. [65] raise the importance of the first mover in the transition towards technological and sustainable models, also analysing the role of the sharing economy. Understanding the needs of consumers allows the creation of new production systems supported by sustainable design and prototypes. These prototypes, aided by the transformation and analysis of real-time data, will improve sustainability by reducing waste [10]. In this sense, Lin [101] points out that the user experience obtained from intelligent products is key to intelligent decision-making and the successful development of new products in the Industry 4.0 environment.

#### 4.2.3 Line 3: the circular economy in the art world

In this third classification, we find a series of articles that highlight the need to integrate the CE into artistic and craft creation [39] to reduce its negative externalities. For this reason, forms of artistic production are sought that use new designs [40], more sustainable designs, or eco-design that feature renewable energy sources, efficient technologies, care for resources, cleaner processes, and less waste generation [41].

The production of CE criteria can lead to the creation of unique and significant works of art, generating impactful pieces that use circular and regenerative materials that can be reintroduced into the system [42] and inspire new business models and sustainable entrepreneurship models [43]. In the articles analysed, we find arguments that reinforce these considerations and examples of practical applications of CE to the world of arts and crafts. Thus, Oliveira et al. [41] study the adoption of CE in companies in the furniture sector, especially in the management of materials and waste. On the other hand, Chavez et al. [98] address the so-called servitization revolution as a generator of income and architect of the product as a service (PaaS), relying on the lean methodology. Continuing with the analysis of technology and circular production processes, they address the high-value practices in manufacturing that are applied in this type of production system. Scholars also highlight the product-service system (PSS), PaaS, predictive maintenance, and remanufacturing and refurbishment, analysing them from the perspective of the business model, considering the value and costs of the implementation of these practices, and highlighting the need for customers to know the value proposition of the company supported in the CE [102]. This is also useful for the maintenance and sustainable conservation of works. Another important issue is the positive message about sustainability sent to society by artists [39].

The benefits of the implementation of the CE are evident, so companies are undertaking transition processes towards this model. This has led to the development of new models such as the circular manufacturing system (CMS) or the product-service system (PSS). The use of an integrated technological platform with predictive capacity allows the implementation of the CMS and PSS [97]. When a company introduces a service that replaces the sale of the product, we find servitization, which drives the creation of PSS and makes production more sustainable than that generated with simple sales and the linear model [91].

Technologies related to data analysis, digital platforms, AI algorithms, the Internet of Things, and blockchain are the most applied solutions in solving problems related to the implementation of the CE. Demestichas & Daskalakis [99] state that the research has paid great attention to the R “reduce”, which unites most of the research. However, technologies applicable to the other “Rs” are also being developed to promote reuse or recycling. Therefore, information technologies are driving forces in the development of the CE model. One of these technologies is fuzzy logic, which models partial truth. AI developments drive the CE, especially in those issues that require data transfer.

Therefore, the idea of design for sustainability arises [15] that opposes the traditional production system (linear economy), establishing located making as a method to understand the development of products, practices, and materials by the principles of sustainability and criteria established by Walker [104]. Another group of relevant articles analyses the development of Industry 4.0 and its implications in the CE [101].

#### 4.2.4 Line 4: relevance of the design stage and training

In art, design occupies a relevant position within the scope of production. The second most destructive industry and the shift towards more sustainable models are slower than expected [40, 106] to analyse the transformation of the textile industry driven by technology and sustainability. The authors state that in the design stage, the entire production process and even the relationship with the consumer must be considered. Sometimes, not knowing the consumer's position on the transformation of products towards more sustainable models, producers may not undertake the changes. That is why it is necessary to connect the demand of the users with the new prototypes [101].

Innovation in materials, technology, and processes must be shared to meet today's global challenges. The work of Pitti [107] highlights how companies have emerged that promote recovered materials, such as wood, offering added value and lower environmental impact on production systems. Design is also a relevant factor in this context of AI and art for Singh & Gu [108], who review five generative design techniques used in architectural design, cellular automata, genetic algorithms, L-systems, form grammars, and swarm intelligence. Based on the literature on design cognition and the recent theoretical work on digital design thinking, there is a need for an integrated generative design framework to improve design exploration support for human designers.

Education can also serve as a driving vehicle for these changes in contemporary artistry. Design education can be recontextualized to generate the social change that society demands today [39] by developing methods and curricula that promote the transformation of processes towards sustainability, considering upcycling and the values of craftsmanship. On the other hand, higher education institutions play a pivotal role in transforming local, national, and global economies, and they will drive change towards the CE in the coming years [105].

## 5 Discussion and conclusions

After the review of the literature, the analysis of the 60 selected articles allowed us to identify the current situation of the incorporation of new technologies, especially AI, into the world of art and to promote its sustainability. Every year, there is a greater development of AI applications, which are also more frequent and accessible for artists and companies in the sector. These applications, base codes, and repositories are generating new opportunities and business models related to the art industry [77]. As a result, new concepts have also appeared, such as assisted art and new platforms and applications for artists, for instance, Playform [55] or Dall-e. The review has also allowed us to identify the algorithms that are being used for the creation of art and the implementation of CE in this industry. Certainly, the contribution of AI applications to CE must be considered from the design stage, integrating sustainability and art with AI applications. The review has revealed the problems generated by applying a technical concept such as AI to a creative concept such as art. We have analysed how this art is perceived by consumers. We can conclude that it is still in its infancy, but technologies have already made a strong impact on the art world, a trend that will grow over time.

## CRediT authorship contribution statement

**Pedro Núñez-Cacho:** Writing – review & editing, Writing – original draft, Validation, Methodology, Formal analysis, Data curation, Conceptualization. **Georgios Mylonas:** Investigation, Formal analysis, Conceptualization. **Athanasios Kalogeras:** Writing – original draft, Supervision, Methodology, Investigation, Formal analysis, Data curation, Conceptualization. **Valentín Molina-Moreno:** Writing – review & editing, Writing – original draft, Investigation, Funding acquisition, Formal analysis.

## Declaration of competing interest

The authors declare no competing interests.
